# Factors Affecting the Functioning of Critical Medical Equipment in a Tertiary Care Hospital

**DOI:** 10.7759/cureus.102926

**Published:** 2026-02-03

**Authors:** Anant Gupta, Nirupam Madaan, Amit Lathwal

**Affiliations:** 1 Hospital Administration, All India Institute of Medical Sciences, New Delhi, New Delhi, IND

**Keywords:** cardiac monitors, critical medical equipment, downtime, infusion pump, ventilators

## Abstract

Objective

In this study, we aimed to estimate the mean downtime of the commonly used equipment and to identify the causes of their downtime in a tertiary care hospital.

Methods

Data were collected through retrospective record analysis of commonly used hospital equipment over two years, followed by prospective data collection on the same equipment over six months to capture key maintenance parameters.

Results

The average downtime index for all equipment was 8.78%. It was observed that cardiac monitors had the maximum downtime at 41.8%. The main causes of downtime were inadequate maintenance at 25.28%, product failure at 14.94%, and mishandling at 14.18%.

Conclusions

It was observed that newer equipment experienced less downtime. A lack of spares and accessory inventory in both hospital stores and with vendors contributed to downtime. Given staff turnover and a lack of reinforcement, personnel operating the equipment were often without adequate operational knowledge. Some good maintenance practices identified included the provision of extra accessories by vendors in accordance with the terms and conditions of the tender. The main causes of downtime were inadequate maintenance, product failure, and mishandling, which together accounted for nearly half of all causes.

## Introduction

Maintenance extends beyond repairing equipment after breakdowns and includes regular upkeep to ensure its continuous availability for clinical use. The acquisition and maintenance of patient care equipment are complicated by multiple factors, such as increasing sophistication, the large number of manufacturers offering similar equipment, limited capital funding, difficulties in setting acquisition priorities within complex healthcare organizations, and insufficient attention to the total cost of ownership, including installation, maintenance, and operation, in addition to the purchase price. Proper maintenance directly affects equipment performance and safety. Poorly maintained equipment is more prone to breakdowns and may cause injury to patients [1]. Similarly, in developing countries, the unavailability of medical equipment due to frequent breakdowns and prolonged repair times is a major constraint to appropriate patient care [2].

The reliability of medical equipment depends on the quality of its design, components, and workmanship. In addition, factors such as adequate user training, the availability of utilities and environmental support, and the timely procurement of appropriate accessories significantly influence equipment downtime [3]. Although equipment breakdown is frequently cited as a cause of poor hospital services, there is a lack of hospital-based studies that systematically examine equipment downtime and its determinants. In developing countries, equipment unavailability due to breakdown continues to be a major constraint to effective patient care. Contrary to common perception, older equipment does not necessarily fail more frequently, as downtime is influenced by maintenance practices rather than age alone. Equipment downtime results in financial losses, patient inconvenience, and reduced confidence in after-sales support. Ensuring that equipment remains functional, accurate, and safe through effective maintenance is therefore essential for delivering high-quality healthcare. In this context, the present study aimed to estimate the mean downtime of commonly used medical equipment and to identify the causes of downtime in a tertiary care hospital.

## Materials and methods

The study was conducted at a tertiary care teaching hospital in New Delhi, one of the pioneer institutions in the country. Data were collected through a two-year retrospective record analysis to assess long-term patterns of equipment downtime, followed by six months of prospective data collection to capture real-time maintenance practices and operational parameters not routinely documented. The differing durations of the two phases were intentionally selected and did not affect the overall interpretation of equipment downtime. Data collection was carried out both from hospital stores, where purchases had been made, and from user areas, including wards and the ICU.

The equipment was selected for the study based on criteria including universal use across all clinical areas, criticality, and a long life cycle of 10 years or more, as specified in the terms and conditions of procurement. Based on these criteria, eight medical equipment items were identified, four high-cost items exceeding 2 lakhs and four low-cost items. Accordingly, ICU beds, defibrillators, ventilators, suction machines, cardiac monitors, syringe infusion pumps, ultrasonic nebulizers, and a resuscitation cart were included in the study. Information was collected using a structured questionnaire. A comprehensive list of the equipment, along with serial numbers, was prepared, and the functionality of each item was assessed simultaneously. The data collected also included the year of installation, the service provider, and the contact details of the service provider.

Retrospective data collection and record analysis (April 2014 to March 2016) of the existing equipment logbook in wards and the service slip provided at the time of breakdown or preventive maintenance by the service engineer, and kept with the sister in charge, was conducted for a period of 24 months. The service slips in the service engineers’ service book were also assessed to determine the downtime of the equipment. Along with the records in wards, there were two sources of data in the hospital store regarding the downtime of equipment. One was the complaint register, which consisted of complaints made by the respective wards to the hospital store when the service provider did not respond to their calls. The second was the official letters issued by the hospital store to the service provider for not providing adequate maintenance of the equipment.

Prospective data collection was conducted for a period of six months in each of the selected wards through weekly ward visits, and additional visits were made whenever the selected equipment broke down. The breakdown date was noted during these visits to calculate the downtime index. Downtime was calculated using calendar days. Any day on which the equipment was reported as non-functional, regardless of expected usage, was counted as downtime. This approach was adopted to reflect the actual availability of equipment in routine hospital operations. Table 1 presents the indices for the downtime of the equipment.

**Table 1:** Indices for equipment downtime BM: breakdown maintenance; PPM: planned preventive maintenance

Indices	Formulae
Loading time	Uptime + downtime
Uptime	Operating time + standby time
Downtime	Time of equipment failures + time of maintenance + time of inspection + time of repair + time of calibration + time accounting for lack of consumables/accessories
Availability	(Uptime/loading time) x 100
Down time indices	(Downtime in hours/service time in hours) x 100
BM index	(Total hours spent in breakdown/total hours available for working) x 100
BM completion rate	(Number of BM completed/number of BM scheduled) x 100
PPM completion rate	(Number of PPM completed/number of PPM scheduled) x 100

To assess the causes of downtime, retrospective data collection and record analysis were performed by reviewing the equipment logbook, service slips (with ward sisters as well as service engineers), store records, complaint registers, and written communication with the vendor. Prospective data collection was conducted for a period of six months in each of the selected wards through weekly ward visits, and additional visits were made whenever the selected equipment broke down. During these visits, the date, equipment name, serial number, and type of call (breakdown or preventive maintenance) were noted, and staff interviews were conducted using a semistructured questionnaire with staff nurses, technicians, and service engineers to ascertain the causes of downtime of medical equipment.

After data collection, Epi-Info version 3.5.4 was used to enter the data, which was then transferred to Microsoft Excel 2010 for data cleaning. Data analysis was performed using Stata 11. The downtime of medical equipment and its causes were analyzed using descriptive statistics and presented as proportions. The Institute Ethics Committee for Post Graduate Research at the All India Institute of Medical Sciences, New Delhi, approved the study (IECPG-53/27.11.2015). Informed written consent was obtained from all participants, including staff, nurses, and technicians who were interviewed.

## Results

Of the 250 pieces of medical equipment, cardiac monitors accounted for the largest proportion (n = 81, 32.4%), followed by infusion pumps (n = 81, 30.0%) (Table 2). The Medicine ward accounted for the highest share of these devices, with 74 (29.6%) and 71 (28.4%), respectively (Table 2).

**Table 2:** Distribution of equipment and their functionality ICU: intensive care unit

Equipment	Total	Percentage
Cardiac monitor	81	33
Defibrillator	11	4
ICU bed	30	12
Infusion pump	75	30
Resuscitation cart	8	3
Suction machine	17	7
Ultrasonic nebulizer	20	8
Ventilator	7	3
Total	250	100
Functional equipment	21	9
Non-functional equipment	228	90.99
In condemnation	1	0.01

Downtime of various medical equipment

Only about 8.4% of equipment (Table 1) was found to be non-functional, and the average downtime index during the 18-month study period was 8.78%. It was observed that downtime for cardiac monitors (41.8%) was the highest, with 19 of the 81 cardiac monitors out of order for almost half of the study period. Additional stratified analyses of cardiac monitor downtime were performed based on equipment age, manufacturer, and ward location. Downtime was substantially higher in older monitors (more than six years) compared to newer devices, largely due to increased product failures and limited availability of spares. Considerable variation was also observed across manufacturers, with certain original equipment manufacturers contributing disproportionately to overall downtime. In contrast, ward location did not demonstrate a consistent association with downtime, suggesting that the observed downtime was primarily related to device-specific and vendor-specific factors rather than systemic ward-level issues.

Formal multivariable analysis was not performed, as the primary objective of the study was descriptive, and the sample size within individual strata was limited. However, stratified analysis yielded meaningful insights into the major contributors to downtime. The downtime of the syringe infusion pumps and resuscitation carts was approximately one-fourth (25%) of the six-month study period. The downtime of the ventilators accounted for 20% of the total days under study (six months) (Table 3).

**Table 3:** Equipment maintenance indices of the studied equipment for the year 2016 BM: breakdown maintenance; PPM: planned preventive maintenance; ICU: intensive care unit

Equipment	Uptime in % (availability %)	Downtime in %	BM completion rate in %	PPM completion rate in %
Cardiac monitor	58.2	41.8	30.9	75.0
Defibrillator	98.8	1.2	100.0	100.0
ICU bed	98.1	1.9	68.8	100.0
Infusion pump	73.3	26.7	42.6	83.3
Resuscitation cart	72.2	27.8	40.0	0.0
Suction machine	98.4	1.6	94.4	100.0
Ultrasonic nebulizer	98.9	1.1	82.4	0.0
Ventilator	81.4	18.6	66.7	100.0

The downtime of cardiac monitors ranged from 6% to 80% across different original equipment manufacturers (OEMs). Newer cardiac monitors, less than six years old, had downtime of under 10%, attributable to fewer product failures and a lower probability of breakdown. It was found that certain equipment continued to function well, but its accessories broke down frequently. One manufacturer provided accessories, such as an oxygen saturation (SpO_2_) probe and a noninvasive blood pressure (NIBP) cuff, as additional items, which the hospital store had purchased according to the terms and conditions of the tender. These accessories were used in case of probe or cuff failure. Another manufacturer provided a replacement monitor while repairs were ongoing, which resulted in reduced downtime. In contrast, a cardiac monitor from one manufacturer failed during the warranty period, preventing negotiation of the comprehensive maintenance contract (CMC) and resulting in 80% downtime. For defibrillators, downtime ranged from 0% to 2.60%, and for ICU beds, downtime ranged from 0% to 3.14%.

The downtime of the syringe infusion pump ranged from 0% to 81%. Syringe infusion pumps purchased after 2015 failed early. Another model exhibited multiple manufacturing defects, which led to the replacement of all syringe infusion pumps in 2015. However, the replacement infusion pumps from the same OEM, procured in 2015, experienced similar breakdown issues. For the resuscitation cart, one OEM had permanently shut down, resulting in the absence of maintenance support. The resuscitation cart was more than 15 years old, and its downtime was approximately 33%. Resuscitation carts from another manufacturer, which were under warranty, had a downtime of 14.2%. The primary reason for downtime was the delay by the service provider in completing repairs. The suction machine had downtime of less than 5%, as it was minimally used, given that suction points were available in almost every ward cubicle. Ventilator downtime ranged from 5% to 36%.

Causes of downtime of various medical equipment

The main causes of downtime were product failure (5.54%), inadequate maintenance (5.47%), and administrative issues (3.62%) (Table 4). The primary reason for product failure was that the syringe infusion pump failed during the warranty period, necessitating replacement with a newer model. Administrative issues were largely related to delays in file approval within the stores department.

**Table 4:** Rate of equipment downtime ICU: intensive care unit

Category	All	All	Cardiac monitor	Defibrillators	ICU beds	Syringe infusion pump	Resuscitation cart	Suction machine	Ultrasonic nebulizer
Inadequate maintenance	5.47	16	0	0.29	0.29	0	0.64	0.44	2.19
Product failure	5.54	2.85	0	1.09	13.59	0	0	0	13.9
Mishandling	3.04	6.66	0	0.2	1.12	14.89	0.42	0	0
Software problem	1.59	1.71	0.34	0	3.08	0	0	0.27	2.5
Administrative issues	3.62	6.65	0	0	4.84	0	0	0	0
Staff not trained	0.13	0.02	0.84	0.18	0.04	0	0.58	0.19	0
Non-availability of spares, accessories, and consumables	2.21	5.66	0	0	1.22	0	0	0	0
Wear and tear	1.95	2.31	0	0.16	2.51	12.98	0	0	0
Environmental stress	0.01	0	0	0	0	0	0	0.16	0
Total	23.56	41.8	1.2	1.9	26.7	27.8	1.6	1.1	18.6

## Discussion

A good maintenance program in an institution or hospital is essential for maintaining medical equipment and ensuring the requisite uptime. Although every hospital aims for 100% uptime, most strive for a more realistic uptime of 95%, allowing for preventive maintenance, calibration, equipment rest, and minor breakdowns. To develop an effective maintenance program, it is necessary to maintain a history sheet for each piece of equipment, containing all relevant equipment details.

Downtime of medical equipment: what really matters

The average downtime was 8.78% over a 30-month period, which was higher than the acceptable 5% as per the terms and conditions of the tender. Downtime was highest for cardiac monitors, with 19 of the 81 monitors out of order for almost half of the study period. Downtime was approximately 25% for syringe infusion pumps and resuscitation carts, and 20% for ventilators.

Several factors were identified as contributing to equipment downtime. It was observed that newer equipment experienced less downtime, consistent with the findings of Mkalaf et al. [4]. However, some equipment, such as syringe infusion pumps and ICU beds, showed high downtime even during the warranty period due to manufacturing defects and early product failures. In these cases, downtime was further prolonged by delayed vendor response times, unavailability of immediate replacement units, and administrative delays in approvals for replacement or escalation, despite the equipment being under warranty. This observation indicates that while most equipment performs reliably during the warranty period, devices with inherent manufacturing defects or early failures may still experience significant downtime if contractual and administrative processes are weak. Ideally, such equipment should be identified during installation and commissioning and replaced promptly to minimize service disruptions.

Another important cause of downtime was administrative issues, involving both vendor and hospital store processes. A key factor identified was weak contract management. Older syringe infusion pumps experienced downtime of more than 50% due to age and difficulty in obtaining spare parts. Consequently, the lack of spares and limited inventory of accessories in both the hospital stores and with the vendor contributed to downtime. In a study by Patil et al. [5], 11 pieces of equipment had downtime of approximately 60 months, and three pieces had downtime of more than three years, primarily due to the absence of a service contract or insufficient repair budget. In contrast, in our study, the main reasons were weak contract management, poor record-keeping, and inadequate coordination within the stores.

Another reason for repeated breakdowns was the human factor, including a lack of training and careless handling by staff, which contributed to equipment downtime. Instances included defibrillator pads not being cleaned of gel, power cords not being properly connected, improper use of syringe infusion pumps, incorrect connection of male and female pins of SpO_2_ probes, improper equipment storage, and software-related issues not addressed by staff. These findings indicate that on-the-job training could reduce equipment breakdowns. Although vendors provided training during equipment installation, no follow-up training was offered during preventive maintenance checks.

Considering staff turnover and lack of reinforcement, personnel operating the equipment often lacked adequate operational knowledge. A study by Tiwari et al. [6] reported an equipment uptime of 95%, attributing the high uptime to complete outsourcing of maintenance. This approach ensured that staff were trained and knowledgeable about the equipment, which also promoted better care of the equipment and improved overall staff satisfaction. Although maintenance in our setting is also outsourced, training is not emphasized. In our study, approximately 40% of observed problems could be resolved through staff training, whereas the remaining 60% of equipment-related issues were due to administrative or logistical deficiencies.

Another cause of downtime was the mishandling of equipment, involving both patients and staff. Agitated patients sometimes pulled on SpO_2_ probes, causing damage. Staff, during ward cleaning, moved ICU beds and syringe infusion pump stands, resulting in physical damage to the equipment. In some cases, the sister in charge did not store the SpO_2_ probe of the cardiac monitor properly, leading to probe breakdown and subsequent downtime. These issues can be prevented through regular staff training. This finding is consistent with the study by Mkalaf et al. [4] in public hospitals in Australia, which reported that approximately 50% of equipment failures were due to human error. Similarly, Srinivasan et al. [7] documented mishandling as a significant cause of downtime, primarily due to insufficient training and a lack of staff sensitization regarding equipment use.

Equipment storage issues - do we need a rethink?

The issue of improper equipment storage, such as stacking monitors that caused display screen breakage and folding sensor leads onto themselves, leading to coagulation in high temperatures, has already been discussed. Additionally, it was observed that some equipment had minimal downtime, not due to good maintenance, but because of limited handling. Users often relied on alternative arrangements for the same function, such as using manifold suction points instead of the suction machine or ultrasonic nebulizers. This resulted in duplication of service, with some equipment lying idle and unused, and therefore never recording any downtime. Overstocking also contributed to underreporting of breakdowns, as broken equipment was replaced by functional units kept in nearby substores, and the failures were never reported. A study by Santos et al. [8] indicated that greater usage of equipment increases the likelihood of failure.

Some good maintenance practices identified included the provision of extra accessories by the vendor, as specified in the terms and conditions of the tender. For this equipment, no downtime was recorded due to administrative issues or lack of spares and accessories. Another vendor provided a replacement unit for equipment that had broken down until the original was repaired. These practices demonstrate that downtime can be minimized by negotiating contracts that include provisions for the temporary replacement of non-functional equipment by the vendor until repairs are completed.

Some firms supplying ICU beds and defibrillators provided prompt service and timely solutions in response to breakdown calls, resulting in minimal downtime. In our study, the better maintenance of defibrillators was attributed to daily checks by the staff nurse and, in some cases, reporting issues before a breakdown occurred, based on experience with the equipment. This aligns with observations by Mkalaf et al. [4] and Ridgway et al. [3], who emphasized that complex and critical equipment require predictive maintenance to maximize uptime. A study by Santos et al. [8] reported 100% uptime for defibrillators, similar to our findings, which was achieved through daily maintenance of critical equipment.

Similarly, Blanch et al. [9] reported negligible downtime for ventilators, attributing the high uptime to predictive maintenance. In many cases, the true causes of downtime were not identified in retrospective record analyses, indicating poor record-keeping and limited awareness of the problem’s extent. A study by McGee et al. [10] found that the cause of failure was unknown in most cases, followed by random failures. In our study, many unknown causes were also not documented in the retrospective data. The gold standard for an effective equipment maintenance program remains a reliable vendor. Vendors with local servicing, readily available spares and accessories, and regular interaction with staff were associated with lower downtime.

Strengths and limitations

One of the main strengths of the study was that data collection was conducted by a single investigator. Since the retrospective component was record-based, the quality of available data varied and was sometimes incomplete. Where gaps or inconsistencies were identified, records were cross-verified using multiple sources, including ward logbooks, service slips, store complaint registers, and vendor documentation. Records that remained incomplete despite cross-verification were excluded from detailed causal analysis but were retained for overall downtime estimation whenever feasible. Compared with the prospective data, downtime recorded in the retrospective records tended to be lower, suggesting that record-keeping was generally poor.

## Conclusions

The study documented that the downtime of medical equipment, including cardiac monitors, syringe infusion pumps, and ventilators, exceeded one-fourth of the total study period. The primary causes of downtime were product failure, inadequate maintenance, and administrative issues. The findings highlight the importance of effective equipment management and underscore a significant gap in medical informatics for proper equipment maintenance. Even in leading medical institutes in India, data on many equipment maintenance parameters is often unavailable. It is recommended that Computerized Maintenance Management System (CMMS) applications be used to record and review equipment logs. The terms of CMC should be modified to ensure equipment is maintained throughout its life cycle. Complex equipment, such as ventilators and defibrillators, should undergo predictive maintenance. Further research on equipment utilization is needed to accurately assess real downtime. Training of the concerned user-area staff should be included in the tender, and installation should be considered complete only when accompanied by satisfactory user training. Adequate storage space should be designated, and proper storage methods should be taught to the sister in charge. In today’s technology-driven healthcare environment, equipment procurement and management represent a significant component of expenditure with a direct impact on patient care. Therefore, good equipment management practices should be a priority for any healthcare facility aiming to deliver cost-effective, high-quality care.
